# Outcomes of I-125 Low-Dose-Rate Brachytherapy in Patients with Localized Prostate Cancer: A Comprehensive Analysis from a Specialized Tertiary Referral Center

**DOI:** 10.3390/jpm14080882

**Published:** 2024-08-21

**Authors:** Philipp Schubert, Vratislav Strnad, Daniel Höfler, Claudia Schweizer, Florian Putz, Michael Lotter, Stephan Kreppner, Andre Karius, Rainer Fietkau, Ricarda Merten

**Affiliations:** 1 Department of Radiation Oncology, Universitätsklinikum Erlangen, Friedrich-Alexander-Universität Erlangen-Nürnberg (FAU), 91054 Erlangen, Germany; vratislav.strnad@uk-erlangen.de (V.S.); andre.karius@uk-erlangen.de (A.K.); rainer.fietkau@uk-erlangen.de (R.F.);; 2 CCC Erlangen-EMN, Comprehensive Cancer Center Erlangen-EMN (CCC ER-EMN), 91054 Erlangen, Germany; 3 CCC WERA: Comprehensive Cancer Center Alliance WERA (CCC WERA), 91054 Erlangen, Germany; 4 BZKF: Bavarian Cancer Research Center (BZKF), 91054 Erlangen, Germany

**Keywords:** prostate cancer, LDR brachytherapy, I-125 seeds, treatment outcomes, low-risk prostate cancer, low-intermediate-risk prostate cancer, patient-reported outcomes, toxicity

## Abstract

Low-dose-rate (LDR) brachytherapy with I-125 seeds is one of the most common primary tumor treatments for low-risk and low-intermediate-risk prostate cancer. This report aimed to present an analysis of single-institution long-term results. We analyzed the treatment outcomes of 119 patients with low- and intermediate-risk prostate cancer treated with LDR brachytherapy at our institution between 2014 and 2020. The analysis focused on biochemical recurrence rates (BRFS), overall survival (OS), cumulative local recurrence rate (CLRR), and the incidence of acute and late toxicities. Patient-reported quality of life measures were also evaluated to provide a holistic view on the treatment’s impact. The median follow-up period was 46 months. CLRR was 3.3% (4/119), five-year BRFS was 87%, and the five-year OS rate was 95%. Dysuria was the most common acute urinary toxicity, reported in 26.0% of patients as grade 1 and 13.4% as grade 2. As a late side effect, 12.6% of patients experienced mild dysuria. Sexual dysfunction persisted in 6.7% of patients as grade 1, 7.5% as grade 2, and 10.0% as grade 3. LDR brachytherapy in patients with prostate cancer is an effective treatment, with favorable clinical outcomes and manageable toxicity. The low CLRR and high OS rates, as well as low incidence of severe side effects, support the continued use of LDR brachytherapy as a primary treatment modality for localized prostate cancer.

## 1. Introduction

Prostate cancer is the second most common cancer and the fifth leading cause of cancer-related death among men worldwide, emphasizing the significant public health impact of this disease. While various treatment modalities are available for localized prostate cancer, selecting the most appropriate therapy requires balancing efficacy, toxicity, and quality of life considerations [1].

Brachytherapy, particularly LDR brachytherapy, has emerged as a highly effective treatment option for patients with localized prostate cancer and found recommendation by numerous guidelines [2,3,4]. This treatment involves implanting radioactive seeds directly into or near the tumor within the prostate gland. The seeds are placed using needles guided by imaging techniques such as ultrasound or CT scans to ensure precise delivery. Planing target volumes include the whole prostate gland in most cases. The radioactive isotope Iodine-125 emits low-energy gamma rays with a half-life of around 60 days. This characteristic allows the seeds to deliver continuous low-dose radiation over several months, focusing the radiation dose on the tumor and prostatic gland while minimizing exposure to surrounding healthy tissues. Several studies have demonstrated the efficacy of LDR brachytherapy, with five-year biochemical recurrence-free survival (BRFS) rates ranging from 85% to 95% [5,6,7,8,9]. Moreover, LDR brachytherapy is associated with a favorable toxicity profile and preservation of quality of life, particularly concerning urinary, bowel, and sexual functions [10].

Despite the proven benefits of LDR brachytherapy, variability in clinical outcomes can occur due to differences in patient selection, treatment planning, and post-therapy follow-up. Additionally, it is important to reflect differences in older and newer studies related to I-125 LDR brachytherapy. Recent studies may be limited by shorter follow-up periods, which might not fully capture long-term outcomes and late-onset side effects. Additionally, advancements in imaging and treatment techniques over time can lead to variability in results, making it challenging to compare recent data with older studies. Older studies may lack detailed patient-reported quality of life measures and modern toxicity grading systems, potentially underestimating the impact of treatment-related side effects. Moreover, changes in patient selection criteria and improvements in brachytherapy protocols can result in differences in treatment efficacy and safety profiles over the years.

This report aims to provide a comprehensive analysis of the clinical outcomes, toxicity, and quality of life in patients with localized prostate cancer treated with LDR brachytherapy at our institution between 2014 and 2020. We retrospectively analyzed the treatment outcomes of 123 patients, focusing on biochemical recurrence rates, overall survival, and incidence of acute and late toxicities. Furthermore, we evaluated patient-reported quality of life measures to provide a holistic view of the treatment’s impact.

## 2. Materials and Methods

### 2.1. Patient Characteristics

We analyzed patients treated with LDR brachytherapy from 2014 to 2020 in our institution. Eligible for our analysis were 119 patients who underwent low-dose-rate (LDR) brachytherapy with I-125 and histologically confirmed low- to intermediate-risk prostate cancer. Additional criteria for inclusion in the analysis were complete digital medical records and sufficient follow-up data The median age of the patients was 69 years (range 48–83 years). Tumor staging revealed that 67 patients (54.5%) had T1c tumors, 41 had T2a, and 6 had T2b. Gleason scores were distributed as follows: 78 patients had a score of 6, and 41 had a score of 7. Within the Gleason 7 cohort, 35 had a score of 7a and 6 had 7b. Prostate-specific antigen (PSA) levels at the time of diagnosis ranged from 0.40 to 19.42 ng/mL, with a median PSA value of 7.09 ng/mL. The majority of patients (85.4%) had PSA levels ≤10 ng/mL, and 13.8% had levels between 10.01 and 20 ng/mL. Further information about patient characteristics is provided in Table 1.

### 2.2. Treatment Characteristics

Prior to brachytherapy, 9 out of 1119 patients underwent transurethral resection (TUR) with a time interval between TUR and brachytherapy ranging from 3 to 204 months. Eleven patients received neoadjuvant androgen deprivation therapy, with a median duration of 5 months (range 2–14 months). The workflow for seed implantation involved several key steps. Initially, a preplanning phase is conducted approximately two weeks before the procedure to determine the necessary activity and quantity of seeds. Shortly before the implantation, an ultrasound-based preliminary plan is created to meet the specific dose volume requirements. The implantation is then performed according to this preliminary plan. Approximately six weeks after the procedure, a CT-based post-plan is conducted to evaluate the results. Further information about patient characteristics is provided in Table 2.

### 2.3. Follow-Up/Statistics

The median follow-up period was 44.5months (range 2–90 months). Three patients (2.4%) were lost to follow-up or died early from other causes. Follow-up included an initial visit six weeks post-brachytherapy for seed verification and dose distribution assessment, followed by a 12-week check-up with PSA measurement and clinical evaluation. Quarterly follow-ups were conducted during the first year, transitioning to biannual evaluations in the second year. Assessments included PSA levels, side effects, and patient-reported quality of life. The analyzed survival outcomes included OS, CLRR, and PSA-free survival. Descriptive statistics and Kaplan–Meier curves were utilized to perform the statistical evaluation using SPSS (IBM Corp. Released 2021. IBM SPSS Statistics for Windows, Version 28.0. Armonk, NY, USA). The log-rank test (Mantel–Cox test) was applied to compare survival curves and determine the statistical significance of differences between groups. Statistical significance was defined at *p* < 0.05. Visualization was performed with GraphPad Prism (version 10.0.0 for Windows, GraphPad Software, Boston, MA, USA).

## 3. Results

### 3.1. Side Effects and Quality of Life

**Figure 1:**
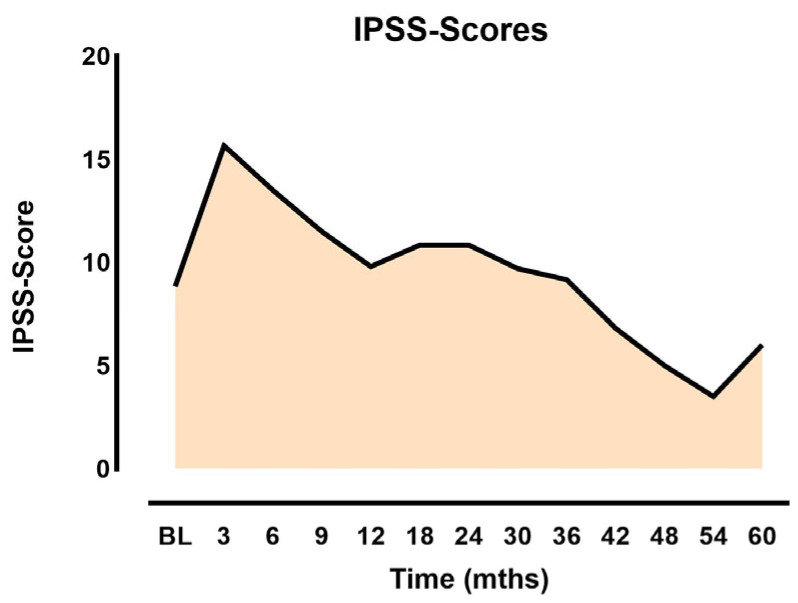
IPSSs as function of time after implantation.

Acute side effects and late side effects were graded according to the fifth version of the CTCAE (Common Terminology Criteria for Adverse Events). Grade 1 acute proctitis was observed in 2/119 patients (2.5%). A total of 117/1119 patients (98.3%) reported no changes from pre-therapy conditions in terms of gastrointestinal side effects. Acute dysuria was absent in 70/119 patients (58.8), 31/119 (26.0%) had grade 1, and 16/119 (13.4%) had grade 2 dysuria. Median International Prostate Symptom Scores (IPSSs) peaked shortly after the procedure to a mean of 15 with a steady decline during the follow-up period (Figure 1).

Acute hematuria was observed in 4/119 patients (3.3%) with grade 1, while 112/119 patients (94.1) had no hematuria. A total of 97/119 did not report any urinary incontinence as an acute transitory side effect, while 13/119 (10.9%) had grade 1, 3 (2.5%) had grade 2, 2/119 (1.6%) had grade 3, and one patient (0.8%) had grade 4 pre-existing complete incontinence. An acute reduction in urine stream was reported by 35/119 patients (29.4%) with grade 1, 14/119 (11.7%) with grade 2, and 1/1190.8%) with grade 3; no acute reduction in urine stream was noted by 64/119 patients (53.8%). Ten patients (8.4%) showed grade 1 post-void residual urine formation, and one (0.8%) had grade 2. No residual urine was reported by 108/119 patients (90.7%). No erectile complaints were reported by 81/119 patients (68.0%).

**Figure 2:**
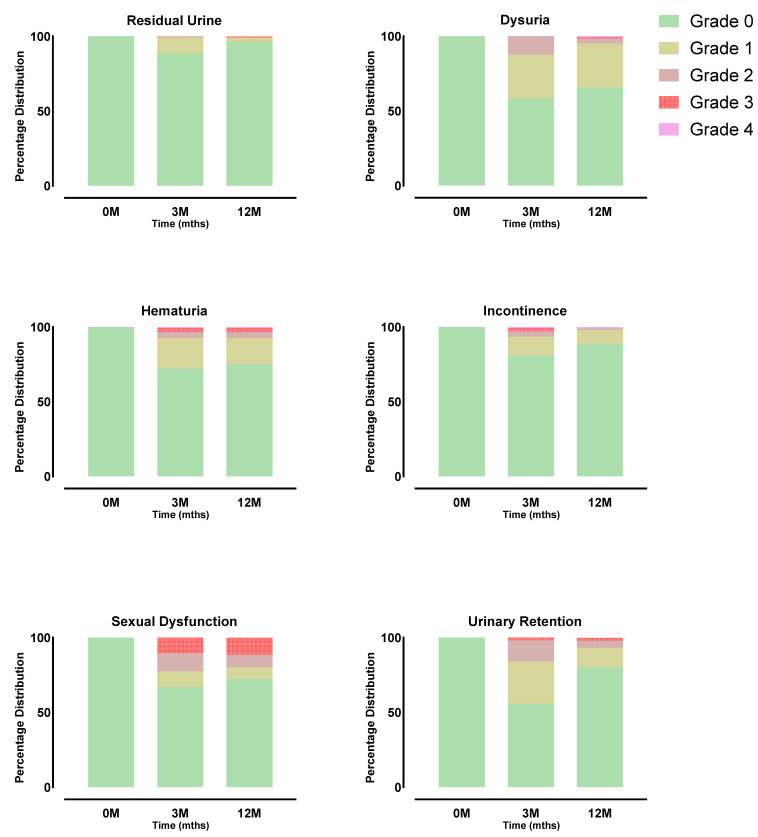
Respective CTCAE toxicity (Grade 0–4) as function of time after implantation.

For late side effects, no data were available for 9/119 patients (7.5%) due to loss to follow-up or death. No cases of late proctitis were reported. Late dysuria was absent in 94/119 patients (78.9%), grade 1 in 15/119 (12.6%), and grade 2 in 1/119 (0.8%). Hematuria was observed in 1/119 patients (0.8%) with grade 1, and one (0.8%) with grade 2. No late hematuria was reported by 108/119 patients (90.75%). Late urinary incontinence was absent in 98/119 patients (82.3%), grade 1 in 10/119 (8.4%), and grades 2 and 4 in one patient each (0.8%). A reduction in urine stream was reported by 16/119 patients (13.4%) with grade 1, 5/119 (4.2%) with grade 2, and 2/119 (1.6%) with grade 3. No reduction in urine stream was reported by 87/119 patients (73.1%). Grade 3 post-void residual urine formation was noted in one patient (0.8%), grade 1 in three (2.5%), and none in 106/119 patients (89.0%). Late sexual dysfunction was reported as grade 1 by 8/119 patients (6.7%), grade 2 by 9/119 (7.5%), and grade 3 by 12/119 (10.0%). No erectile dysfunction was reported by 80/119 patients (67.2%). The frequencies of reported toxicities are depicted in Figure 2.

Patients rated their quality of life (according to the EORTC quality of life questionnaire, question 29) on a scale from 1 (very poor) to 7 (very good). Before therapy, the average score was 5.31 (range 1–7). Three months post-therapy, the average score was 4.95 (range 2–7), and twelve months post-therapy, the average score was 6.0 (range 2–7).

### 3.2. Efficacy

**Figure 3:**
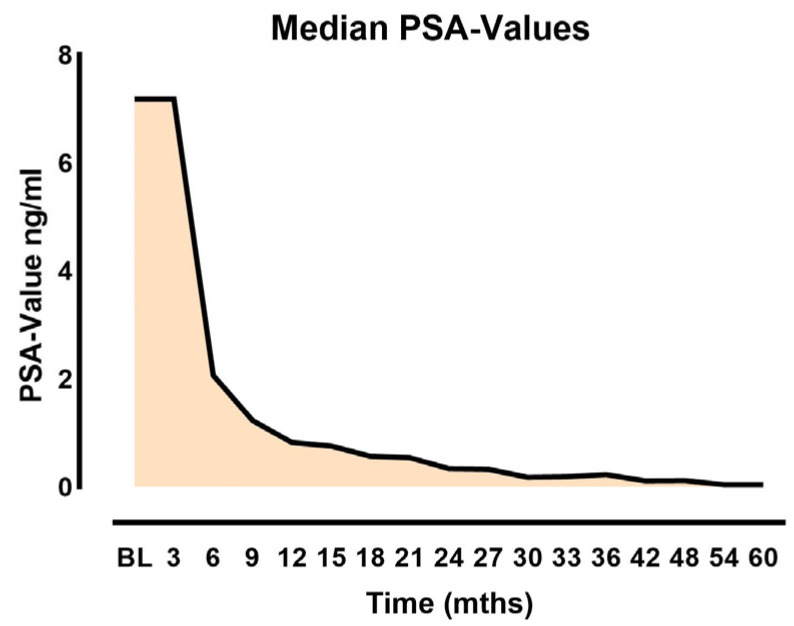
**Figure 2** PSA values as function of time after implantation.

The baseline PSA prior to seed implantation showed a median of 7.18 ng/mL (range 0.79–20.97 ng/mL). The PSA nadir ranged from 0.00 to 4.59 ng/mL, with an average value of 0.42 ng/mL in the studied patient cohort (Figure 3).

**Figure 4:**
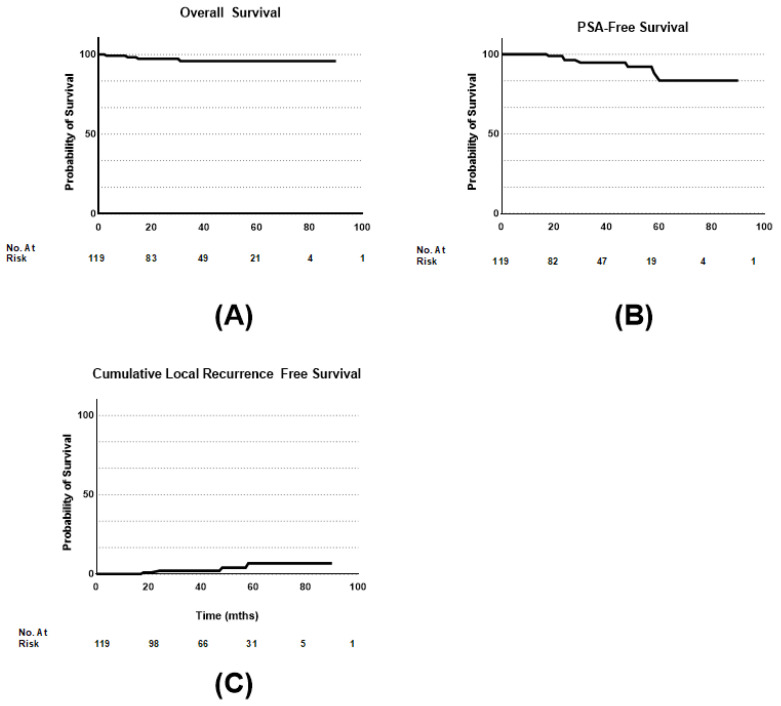
Survival as function of time after implantation. (**A**) Overall survival; (**B**) PSA-free survival; (**C**) cumulative local recurrence-free survival.

In total, 5.8% (7/119) of patients had biochemical recurrence according to the phoenix criteria (>2 ng/mL). However, we documented in total 8/119 patients with PSA recurrences; one patient experienced an increase in PSA in two consecutive measurements, leading to a PSMA-PET-CT scan where a PSMA-positive lymph node was diagnosed. At the time of examination, the patient’s PSA value was only 0.2 ng/mL above the nadir. Due to this finding on the PSMA-PET-CT scan, we classified the case also as PSA recurrence. Thus, 6.7% (8/119) suffered a PSA recurrence. Accordingly, the biochemical recurrence-free 3- and 5-year survival rates are 97% and 87%, respectively. In four of the eight cases of PSA recurrence described above, local recurrence was observed. The local recurrence was confirmed via PSMA-PET-CT. Overall, 3.3% (4/119) of the patients experienced local recurrence, which corresponds to 3-year- and 5-year-cumulative local recurrence rates of 1.6% (2/119) and 3.3% (4/119), respectively. The 5-year survival rate for the whole patient population was 97%. When considering overall survival, it can be noted that all five deaths that occurred during the observation period were not prostate cancer-related. Kaplan–Meier curves are summarized in Figure 4.

For further detailed analysis, we divided the patients into four groups regarding PSA nadir values: group 1 with a PSA nadir value of <0.07 ng/mL with 27/119 patients (22.6%); group 2 with nadir values of 0.07–1.0 ng/mL—72/119 (60.5%), group 3 nadir values between 1.0 and 2.0 ng/mL—13/119 (10.9%), and group 4 with nadir >2.0 ng/mL—7/119 patients (5.8%). 

**Figure 5:**
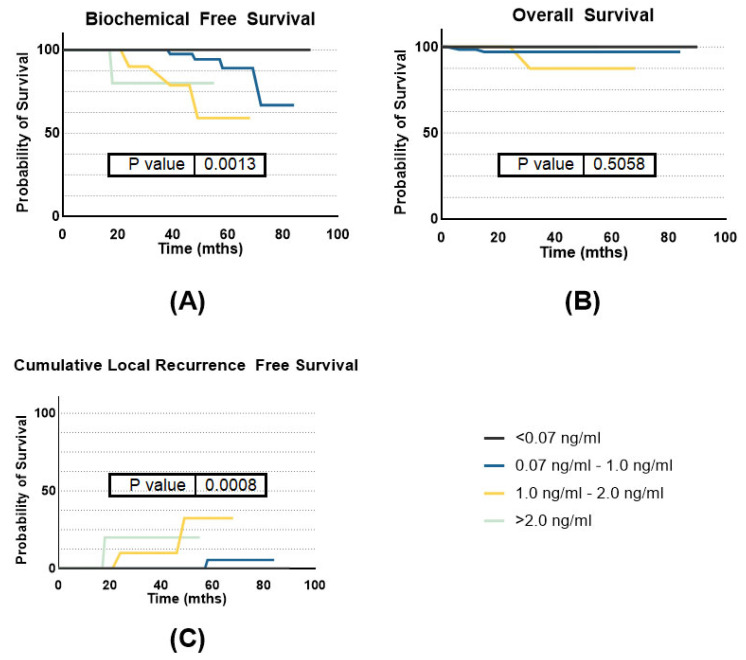
Survival separated by PSA nadir group implantation. (**A**) Biochemical-free survival; (**B**) overall survival; (**C**) cumulative local recurrence-free survival.

At 3 years, BFS rates were uniformly high across groups 1 and 2, both achieving 100%. Groups 3 and 4 had BFS rates of 90% and 80%, respectively. By 5 years, group 1 maintained a BFS rate of 100%, while group 2 had 90%. However, groups 3 and 4 dropped significantly to 59% and 80% (Figure 2). CLRR rates for group 1 were consistently 0% at both 3 and 5 years. Group 2 also maintained 0% at 3 years, slightly increasing to 5% at 5 years. Group 3 showed 10% at 3 years, increasing to 32.5% at 5 years, while group 4 had stable CLRR rates of 20% at both time points. For OS, group 1 exhibited a survival rate of 100% at both 3 and 5 years. Group 2 had a high survival rate of 97.18% at both 3 and 5 years. Group 3 showed OS of 87.5 at 3 and 5 years. Group 4 maintained a consistent OS rate of 100% at both intervals. We found statistically significant differences between the groups for BFS and CLRR (*p* = 0.0013 and *p* = 0.008 respectively). Kaplan–Meier curves are summarized in Figure 5.

Finally, we analyzed subgroups according to the initial risk classification by D’Amico (low risk, 75 patients; low-intermediate risk, 34 patients; high-intermediate, 10 patients) and evaluated efficacy separately for low- and low-intermediate-risk patients (LOW) as well as for high-intermediate- and high-risk patients (HIGH).

**Figure 6:**
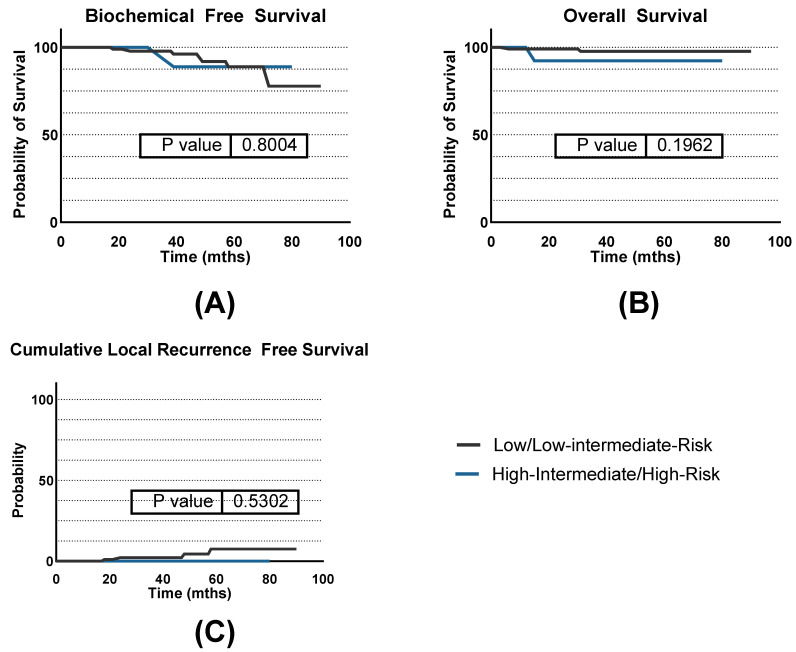
Survival separated by D’Amico risk classification. (**A**) Biochemical-free survival; (**B**) overall survival; (**C**) cumulative local recurrence-free survival.

BFS rates for LOW patients were 98% at 3 years and 89% at 5 years. HIGH patients maintained 100% BFS rates at 3 years and 88% at a 5-year interval. CLRR rates for LOW patients were 2% at 3 years and 7% at 5 years. HIGH patients maintained 0% at 3 and 5 years. Regarding OS, the 3-year and 5-year survival rates for LOW patients were 98%, while HIGH patients had 92% OS rates at 3 and 5 years. No statistical significance was observed comparing the outcomes of the groups (*p* = 0.5302). Kaplan–Meier curves are summarized in Figure 6.

## 4. Discussion

With our analysis, we aimed to contribute recent data on outcomes following LDR brachytherapy with I-125 seeds for low- and intermediate-risk prostate cancer patients. Furthermore, we aimed to provide insights on and the incidence of acute and late toxicities, as well as patient-reported quality of life measures to provide a comprehensive assessment of the treatment’s impact. The results of this analysis demonstrate once again that low-dose-rate (LDR) brachytherapy is an effective treatment for localized prostate cancer. Our cohort of 123 patients, treated between 2014 and 2020, showed good outcomes with a biochemical recurrence-free survival (BRFS) rate in the range of 90% at five years and an overall survival (OS) rate in the range of 95% at both three and five years. These outcomes are consistent with the existing literature, which reports five-year BRFS rates ranging from 85% to 95% for patients undergoing LDR brachytherapy for localized prostate cancer [5,9,11,12].

The toxicity profile observed in our study indicates that LDR brachytherapy is well-tolerated with manageable side effects. Acute urinary toxicity was reported in 26.0% of patients, with dysuria being the most common symptom. At the 12-month follow-up, the incidence of dysuria had decreased significantly, with only 12.6% of patients experiencing mild symptoms. These findings align with previous studies that report acute urinary toxicity rates between 10% and 30% and a major reduction in symptoms within the first year post-treatment [12,13,14,15]. Further late urinary toxicities were relatively low, with only 1.6% of patients experiencing grade 2 hematuria and a negligible incidence of higher-grade toxicities.

Importantly, the incidence of sexual dysfunction was equal to or better than reported rates in other studies, with 67.2% of patients reporting no erectile dysfunction one year post-treatment [12,15,16]. This preservation of sexual function underscores the suitability of LDR brachytherapy for patients concerned about quality of life post-treatment, and especially in younger, sexually active patients.

The recurrence rates observed in our cohort were low, with only 6–7% of patients experiencing PSA recurrence and 3% experiencing local recurrence within five years. These results are particularly noteworthy given that our cohort included a significant proportion of intermediate-risk patients (47.2%). Furthermore, our findings when stratifying patients according to PSA nadir and initial D’Amico classification stress the importance of patient selection for upfront therapy and highlight the predictive value of PSA nadir levels in determining long-term outcomes for prostate cancer patients. Additionally, PSA nadir values may be instrumental in guiding follow-up care for low-risk prostate cancer patients [17]. The utilization of PSMA-PET-CT scans for early detection of recurrences likely contributed to the prompt identification and management of recurrences, explaining the high survival rates observed. However, these diagnostic procedures are not universally available, necessitating careful selection criteria to avoid unnecessary examinations [18].

When comparing our findings to other treatment modalities, such as external beam radiotherapy (EBRT) and radical prostatectomy, LDR brachytherapy offers comparable outcomes with a more favorable side effect profile. For example, EBRT studies report five-year BRFS ranging from 75 to 99% depending on initial risk classifications. These studies also included proton therapy, which offers advantages in terms of dose gradient. Despite comparable effectivity, higher incidences of gastrointestinal toxicities compared to LDR brachytherapy were reported [19,20,21]. Furthermore, the minimally invasive nature of brachytherapy results in shorter recovery times and fewer disruptions to patients’ daily lives, which is a significant advantage over surgical options [10]. Particularly in preserving sexual function and reducing the risk of urinary incontinence, LDR brachytherapy offers significant advantages over radical prostatectomy. Patients undergoing LDR brachytherapy experience lower rates of erectile dysfunction compared to those who have had a prostatectomy, as it is possible to spare surrounding nerves and tissues crucial for sexual function. Additionally, the risk of urinary incontinence is markedly lower with brachytherapy, as this minimally invasive procedure avoids the extensive disruption and damage to anatomical structures relevant for urinary continence such as urinary sphincters.

However, there are several limitations of our study to consider. The retrospective nature of the study may introduce selection bias, and the relatively small sample size limits the generalizability of the findings. Especially in the case of subgroup analysis, it is difficult to draw conclusions due to small sample sizes. Additionally, the follow-up period, while sufficient to capture early outcomes, may not fully reflect long-term toxicities and survival rates. This is especially important for prostate cancer patients since disease progression can be slow, particularly in low-risk situations compared to higher risk patients [22]. In this light, it must also be noted that all deaths that occurred during our examination period were not prostate cancer-related. The endpoint of OS needs to be viewed critically in this situation.

Future research should focus on long-term outcomes of LDR brachytherapy, particularly in high-risk patient populations. Comparative studies with modern imaging and radiotherapy techniques, such as PSMA-PET scans, functional MRI scans, stereotactic body radiotherapy (SBRT), and proton therapy, could provide valuable insights into optimizing treatment protocols [23,24]. Additionally, exploring the impact of focal therapies could further refine patient management strategies [25].

## 5. Conclusions

In conclusion, this study reinforces the efficacy and safety of LDR brachytherapy for localized prostate cancer. The favorable clinical outcomes, low toxicity rates, and positive quality of life metrics support its continued use as a primary treatment modality. Further research with larger cohorts and extended follow-up is warranted to validate these findings and explore potential advancements in brachytherapy techniques.

## Figures and Tables

**Table 1:** Patient characteristics.

Characteristic	Value
Median Age (years)	69.0 (48–83)
Gleason Score 6	79
Gleason Score 7a	37
Gleason Score 7b	6
Mean PSA at Diagnosis (ng/mL)	7.09
PSA Range (ng/mL)	0.4–19.42
cT1a	4
cT1b	1
cT1c	67
cT2a	1
cT2b	6
Neoadjuvant Androgen deprivation (yes)	11

**Table 2:** Treatment characteristics.

Characteristic	Value (Range)
Median Number of Seeds Implanted	53 (49–74)
Median Prostate Volume (cc)	27.4 (9.9–69.5)
Median Dose to 30cc of Urethra (Gy)	129.3 (106.3–148.6)
Median Percentage of Prostate Volume Receiving 100% of the Prescribed Dose (V100)	96.7% (79.68–99.39%)
Median Percentage of Prostate Volume Receiving 100% of the Prescribed Dose (V100) in POST-PLAN	94.0% (57.2–99.71%)
Median Percentage of Prostate Volume Receiving 150% of the Prescribed Dose (V150)	60.4% (42.4–73.06%)
Median Dose to 90% of Prostate Volume (Gy)	116.15 (105.3–127.59)
Median Dose to 2cc of Rectum (Gy)	75.1 (29.5–91.29)
Median Dose to 2cc of Rectum (Gy) in POST-PLAN	58.3 (28.54–91.39)
Dose to 2cc of Bladder (Gy)	70.28 (33.9–112.83)

## Data Availability

The datasets generated during and/or analysed during the current study are not publicly available due to data privacy restrictions but are available from the corresponding author on reasonable request.
